# Practical Notes on Diagnosis and Treatment

**Published:** 1907-11-02

**Authors:** 


### PRACTICAL NOTES ON DIAGNOSIS AND TREATMENT.

### Chorea.

Camphor monobromate has been used successfully in this disease. It was given in capsules, the dose being 0.2 gram. Two doses were given daily, and every other day an additional dose was added until nine were given in the twenty-four hours. In the course of a week, though the case was an extreme one, the patient was able to sit up in bed.

### Pericardial Lesions.

Murmurs or quasi-murmurs, which seem to be prolongations of the first sound, are sometimes heard over the præcordium, and especially over the base and the right ventricle; they are of no prognostic importance. They probably depend on a patch of thickened pericardium, the remains of an extinct pericarditis.—*Sir Wm. Gairdner.*

### Pulsating Vessels in Pharyngeal Wall.

These have been occasionally observed, the patients for the most part being old people. An enlarged ascending pharyngeal, and an abnormal vertebral artery, have been suggested as the explanation. But in four cases reported by Dr. Brown Kelly the author contends that the pulsation was due to a tortuous condition of the internal carotid arteries.

### Enema Rashes.

These are not very uncommon. They are usually erythematous and resemble the eruption of scarlet fever; occasionally they show an urticarial character. For the most part they appear on the buttocks and spread both downwards over the thigh and upwards on the trunk; sometimes they affect the upper limbs and rarely the face. They seldom persist for longer than twenty-four hours.

